# Current status of cancer education in developing and developed countries: identifying the disparities and bridging the gap

**DOI:** 10.3389/fpubh.2025.1608525

**Published:** 2025-08-13

**Authors:** Gulimire Yilihamu, Shalayiding Aierxiding

**Affiliations:** ^1^First Affiliated Hospital of Xinjiang Medical University, Ürümqi, China; ^2^Sixth Affiliated Hospital of Xinjiang Medical University, Ürümqi, China

**Keywords:** cancer education, developing countries, developed countries, disparities, policy support, public awareness

## Abstract

Cancer constitutes a significant public health issue globally. according to the World Health Organization (WHO), cancer is the second most common cause of mortality worldwide, accounting for 10% of all deaths in 2020–2022. GLOBOCAN 2020 data from the International Agency for Research on Cancer (IARC) show that age-standardized incidence rates (ASR) in developed nations are markedly higher than those in developing countries. For instance, Australia reports an ASR of 468.0 per 100,000, while India’s ASR stands at only 97.1 per 100,000. This discrepancy can be attributed partly to more robust cancer registration systems, extensive screening programs, and cancer education prevalent in developed countries. Notably, the participation rate in breast cancer screening in the United States surpasses 70%, in contrast to less than 20% in certain African regions, as reported by the National Cancer Institute (NCI). Through a systematic review, this paper examines the gaps in cancer education policies, resource allocation, educational approaches, public awareness, and healthcare system support between developed and developing countries, proposing strategies to bridge these gaps. The research reveals that developed countries boast well-established policy support, ample financial investment, and advanced educational technologies in cancer education. In contrast, with a later start, developing countries face challenges such as resource scarcity, incomplete policies, and insufficient public awareness. To narrow these disparities, developing countries must strengthen policy support, increase financial investment, particularly in rural areas, improve cancer prevention and control legislation, enhance the accessibility and quality of cancer education, promote innovative educational methods, and elevate public awareness of cancer prevention and control.

## 1 Introduction

A new study by researchers found that in 2022, nearly 20 million new cases of cancer were reported, alongside approximately 9.7 million cancer-related deaths (1). Current estimates suggest that roughly one in five individuals will develop cancer during their lifetime, with mortality rates indicating that about one in nine men and one in twelve women are likely to succumb to the disease (2). As the world approaches the second quarter of the twenty-first century, cancer persists as a predominant contributor to global mortality (3). Demographic predictions suggest that the number of new cancer cases will reach 35 million by 2050 (4). In 2021, Global Cancer Statistics ranked cancer as the leading or secondary cause of death in 127 countries, with it being the third or fourth leading cause in an additional 57 countries (5). This alarming scenario positions cancer as a significant global health threat, notably in developing nations where incidence and mortality rates are surging (6). Over the past decades, there has been a consistent upward trend in both cancer incidence and mortality across various regions and countries (7). The reason for this rise can be attributed to several factors, including population aging, growth, accelerated socioeconomic development, and shifts in risk factors. These dynamics have rendered cancer a principal contributor to reduced life expectancy in many countries (8).

Addressing this escalating health crisis necessitates the enhancement of health literacy among cancer patients, a strategy deemed pivotal for managing the growing cancer burden (9). While substantial advancements have been made in cancer prevention and control within developed countries, many developing nations continue to grapple with significant challenges pertaining to cancer education (10). As the complexity of cancer treatment protocols and healthcare systems intensifies, patients often encounter significant obstacles in navigating their disease journey (11). A well-structured cancer education framework empowers patients, equipping them with the necessary knowledge to fully understand their conditions, communicate effectively with healthcare providers, and actively participate in their treatment regimens. Such empowerment is essential not only for improving health-related quality of life but also for enhancing overall health outcomes among cancer patients.

Cancer education plays a vital role in the landscape of cancer prevention and control, with the potential to significantly lower incidence and mortality rates by fostering public awareness regarding prevention, early detection, and timely treatment (12). This article seeks to systematically review the current state of cancer education across developed and developing countries, analyze the disparities between these regions, and propose strategies to bridge these educational gaps. Through targeted interventions and collaborative efforts, we can enhance cancer education, ultimately improving outcomes for patients worldwide.

## 2 Methods

This systematic review was conducted following the Preferred Reporting Items for Systematic Reviews and Meta-Analyses (PRISMA) guidelines to ensure methodological transparency and reproducibility (13). The search strategy outlined below was designed to identify studies pertinent to the primary research questions regarding the current landscape of cancer education in both developing and developed nations.

### 2.1 Search strategy

**Figure 1:**
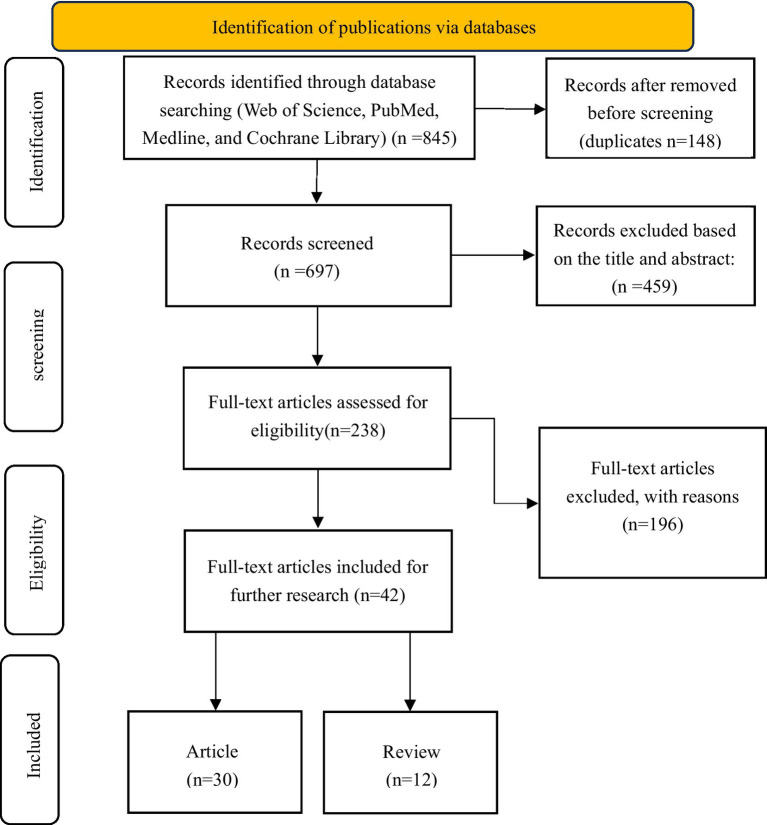
PRISMA flow diagram illustrating the study identification, screening, eligibility, and inclusion process.

A comprehensive systematic search was performed across various electronic databases, including Web of Science Core Collection (WOSCC), PubMed, Medline, and Cochrane Library databases, to identify studies related to the current status of cancer education in developing and developed countries, to identify the disparities, and to bridge the gap. An extensive search was conducted using advanced search capabilities, incorporating key subject terms such as TS = (“cancer education” OR “oncology education” OR “cancer literacy” OR “cancer awareness”) AND TS = (“developing country” OR “low- and middle-income countries” OR “LMIC” OR “low-income country” OR “developed country” OR “high-income country” OR “HIC”). Language restrictions were applied to English, aiming to extract relevant articles published between the years 2004 and 2024, with the data aggregation process concluding on July 21, 2025 (Figure 1).

### 2.2 Selection criteria

In this study, the authors identified eligible publications utilizing a standardized data extraction form to maintain consistency and reliability across the selected databases. The extracted literature was managed using EndNote 20.0 software. Only full-text articles and review articles that met eligibility criteria and contained sufficient information for extraction were included. The initial data retrieval was conducted independently by two researchers, who subsequently discussed and reconciled any discrepancies that arose. The final concordance value reached was 0.90, indicating a substantial level of agreement between the researchers. A comprehensive collection of publications relevant to cancer education in both developing and developed countries has been assembled.

#### 2.2.1 Inclusion criteria

Relevant articles and reviews from the WOSCC PubMed, Medline, and Cochrane Library database focused on the current status of cancer education in developing and developed countries were published between January 1st, 2004, and December 31st, 2024.

#### 2.2.2 Exclusion criteria

(1) Duplicate literature; (2) Notifications, comments, translations, conference proceedings, abstracts, newspapers, patents, news, lectures, autobiographies, etc.

### 2.3 Study selection process

The systematic literature review followed PRISMA guidelines to ensure methodological rigor. Initial searches across Web of Science Core Collection (WOSCC), PubMed, Medline, and Cochrane Library yielded 845 records. After removing 148 duplicates, 697 unique records underwent title/abstract screening, excluding 459 irrelevant studies. The remaining 238 full-text articles were assessed for eligibility, with 199 excluded primarily due to: (1) irrelevance to cancer education (*n* = 124, 63.3%), (2) insufficient comparative data between developed and developing nations (*n* = 53, 27.0%), and (3) inadequate methodological quality (*n* = 19, 9.7%). The final synthesis included 42 studies (30 articles and 12 reviews) that met all inclusion criteria. This selection process, documented through the PRISMA flow diagram (Figure 1).

### 2.4 Quality assessment

We assessed the quality of the articles using the Newcastle–Ottawa scale assessment form for cohort studies (NOS) (14). The NOS form assesses the quality of non-randomized studies through a rating system that allows the assessor to define relevant factors to control for in the given context.

The scale consists of 8 criteria divided into 3 domains: selection, comparability, and outcome. In total, an article can be given 8 stars, with 6 stars or more indicating good quality. In accordance with the Cochrane model (15), 2 reviewers independently assessed the quality of each paper. Consensus was reached through discussion and consultation with a third party. For the domain selection, studies receive credit (a star) for (1) discussion of more than one country representing LMICs or HICs, (2) discussion of more than two countries representing LMICs or HICs (3) discussion of more than one country representing LMICs and HICs, and (4) demonstration that the outcome of interest was not present at baseline. The last criterion is not applicable to the studies in this literature review and was thus not considered. For comparability, credit is given for (5) comparability of cohorts based on design or analysis. Studies were given 1 star if they only discussed cancer education in developing or developed nations, and another star if they discussed both nations. For the domain outcomes, studies can receive 3 stars, for (6) assessment of outcomes, (7) follow-up long enough for the changes in the cancer literacy to occur, and (8) adequate follow-up of the cohorts. Follow-ups of approximately 6 months or longer were deemed adequate.

The quality assessment of the 42 included studies revealed that 36 studies (85.7%) were rated as good quality (≥6 stars), while 6 studies (14.3%) were rated as poor quality (<6 stars). The good quality studies demonstrated robust methodology across all domains, with particularly strong performance in comparability. The poor quality studies exhibited common limitations including high attrition rates (*n* = 3), inadequate control groups (*n* = 4), and restricted geographic scope (*n* = 3). Review articles tended to score lower in comparability when focusing exclusively on either LMICs or HICs. Intervention studies generally performed better in outcome assessment due to their longitudinal designs. The overall findings indicate that while most studies met good quality standards, persistent methodological challenges remain in cancer education research, particularly concerning long-term follow-up and balanced representation of different economic settings, which should be addressed in future studies to strengthen the evidence base (Table 1).

**Table 1:** Quality assessment based on the Newcastle Ottawa Scale.

Article	Selection (Max 3*)	Comparability (Max 2*)	Outcome (Max 3*)	Total quality
Wu et al. (6)	***	*	**	Good
Al-Hasan et al. (9)	***	**	**	Good
Freeman (10)	*	**	*	Poor
Agbedinu et al. (11)	**	**	**	Good
Bray et al.(12)	***	**	**	Good
Karasiewicz et al. (16)	**	*	***	Good
Ramsey et al. (20)	**	*	*	Poor
Carter-Bawa et al. (21)	**	**	**	Good
Barros et al. (24)	***	**	***	Good
Amgad et al. (25)	**	**	***	Good
Wagner et al. (28)	***	**	***	Good
Silva et al. (29)	***	**	***	Good
Haileselassie et al. (30)	***	**	**	Good
Sharma et al. (31)	***	**	***	Good
Bamodu and Chung (33)	***	*	***	Good
Vilar-Compte et al. (34)	**	**	*	Poor
Pimple and Mishra(35)	***	**	***	Good
Malik and Kaplan (36)	**	**	**	Good
Zubizarreta et al. (37)	***	**	***	Good
Sharma et al. (38)	***	**	***	Good
Ramirez and Thompson (39)	**	*	**	Poor
Macciotta et al. (40)	***	**	***	Good
Patel et al. (42)	***	**	***	Good
Sauvaget et al. (43)	*	**	**	Poor
Ige et al. (45)	***	**	***	Good
Mbanda et al. (46)	***	**	***	Good
Abdel-Wahab et al. (47)	***	**	***	Good
Kante and Målqvist (48)	***	**	***	Good
Hodge et al. (51)	***	**	***	Good
Barragan-Carillo et al. (53)	***	**	***	Good
Dee et al. (54)	***	**	***	Good
Zhu et al. (55)	***	**	***	Good
Makadzange et al. (57)	***	**	***	Good
Weeks et al. (58)	**	**	***	Good
Dartibale et al.(59)	***	**	**	Good
Richards et al. (60)	***	**	***	Good
Hamashima and Takahashi (63)	**	*	**	Poor
Karlsson et al. (66)	**	**	***	Good
Cabanes et al. (68)	***	*	***	Good
Elmore et al. (69)	***	**	***	Good
Okoroafor and Christmals (72)	**	**	***	Good
Shaffer et al. (73)	***	**	**	Good

Stars-system is explained in detail in Section 2.4 Quality assessment.

## 3 Results

### 3.1 Current status and disparities of cancer education in developed and developing countries

Cancer has become one of the primary threats to human life and public health, both in developed and developing countries. According to the WHO’s “2020 Global Cancer Report,” in 2018, there were 19.29 million new cancer cases and 9.96 million cancer-related deaths globally, with the world’s largest developing country China accounting for 4.57 million new cases (23.7% of the global total) and 3 million deaths (over 30% of the worldwide total) (16, 17). However, the International Union Against Cancer surveyed public awareness of cancer, revealing that 20% of respondents had no understanding of cancer, while 40% had some knowledge but still feared it. It turns out that fear of cancer stems from a lack of correct understanding, highlighting the critical importance of cancer education in enhancing public awareness (18).

To reduce cancer incidence and mortality rates, countries worldwide have gradually incorporated cancer education into national cancer control strategies or plans, promoting it to the entire population (19). In developed countries, for instance, the United Kingdom has integrated cancer education into the national curriculum for primary and secondary school health education (20). The United States has focused on disseminating core knowledge about cancer prevention and control among adolescents (21, 22). Australia, with the highest rate of cervical cancer prevention knowledge dissemination, has been promoting HPV vaccination and screening since the 1990s, achieving significant results over the past three decades. At this rate, Australia is expected to achieve the WHO’s target of less than 4 cases per 100,000 people annually by 2028 (23). Portuguese scholars have developed a new method for cancer prevention education by training 54 high school biology teachers in “cancer prevention education,” enhancing their knowledge and then using them as intermediaries to disseminate core information to 5,000 students. The evaluation revealed that 90% of the teachers demonstrated significant improvement in their cancer literacy, and their enthusiasm and proficiency in disseminating core knowledge effectively reached over 70% of the students (24).

However, the situation of cancer education in developing countries could be different. Compared to developed countries in Europe and the Americas, developing countries have initiated cancer education later and have yet to establish mechanisms for the universal promotion and dissemination of cancer education (25). Most efforts focus on high-risk groups and regions, with gaps in the dissemination of concepts and their effective implementation. In recent years, however, developing countries have increasingly recognized the importance of cancer education. For example, in 2019, the Health China Action Promotion Committee issued the “Health China Action (2019–2030),” setting targets for nationwide awareness of core cancer prevention knowledge to reach at least 70% by 2022 and 80% by 2030 (26, 27). Peer-led initiatives, such as Uganda’s Cervical Cancer Network, significantly boosted screening rates by 40% through the training of community health workers (28). School-based interventions, exemplified by Brazil’s Adolescent Health Program, achieved an impressive 85% acceptance rate for the HPV vaccine by integrating educational content into school curricula (29). Culturally tailored materials, such as visual pamphlets from Ethiopia, doubled participation in screening programs (30). Additionally, public-private partnerships, like Zambia’s Pink Ribbon Red Ribbon, engaged 70% of the population in cervical cancer initiatives (31). Countries like Grenada have developed cervical cancer control plans and implementation guidelines, emphasizing the need to strengthen cancer education to foster public awareness among women and encourage participation in screening programs (32). These approaches emphasize the significance of localized, scalable strategies to enhance cancer literacy and promote early detection in low- and middle-income countries (LMICs).

Globally, with significant disparities in education and awareness between developed and developing nations, cancer remains a critical public health challenge. While developed countries have implemented comprehensive cancer education programs through school curricula and national campaigns, developing countries are still establishing systematic approaches, often focusing on high-risk populations. Recent initiatives in LMICs demonstrate that peer-led education, school-based programs, culturally adapted materials, and public-private partnerships can effectively enhance cancer literacy and screening participation. Moving forward, scaling up culturally appropriate and locally adaptable education models will be crucial for improving cancer prevention and early detection worldwide.

### 3.2 Policy support and funding

In LMICs, cancer education and prevention face significant challenges due to fragmented policy frameworks and inadequate funding, with most countries lacking comprehensive national cancer control plans (NCCPs) that explicitly integrate education and prevention strategies (33). Competing health priorities, such as infectious disease programs, often divert limited resources, resulting in minimal budgetary allocations for cancer-specific initiatives. Studies showed that LMICs spend less than $10 per capita on cancer education compared to $150–300 in high-income countries (HICs) (34, 35). Additionally, bureaucratic procurement processes and reliance on external aid further hinder sustainable progress (36). Despite these barriers, some LMICs have demonstrated success through policy innovations, such as Rwanda’s mandated workplace and community education programs, and Zambia’s government-backed radiotherapy training initiatives (37). Public-private partnerships (e.g., Nigeria’s loan-based equipment procurement) and task-shifting models (e.g., Uganda’s community health worker training) offer cost-effective solutions to improve cancer literacy and early detection (38).

In HICs, cancer education and prevention benefit from robust policy frameworks and substantial funding allocations, with comprehensive NCCPs integrating education as a core component, exemplified by the U.S. National Cancer Act (1971) and subsequent initiatives like the Cancer Moonshot, which allocated $1.8 billion annually for research and public awareness campaigns (39). Government-funded institutions such as the NCI in the U.S. and Cancer Research UK provide structured curricula for schools and communities, leveraging digital platforms and mass media to achieve public awareness rates exceeding 70% for screening programs like mammography (40). Policy coherence is reinforced by legislation mandating cancer education in national health strategies, as seen in Japan’s Basic Law on Cancer Control (2006), which earmarks dedicated budgets for prevention and early detection (41). Funding stability is ensured through multi-year budgetary commitments, enabling advanced technologies (e.g., HPV vaccination programs with >80% coverage in Australia) and workforce training (42). Public-private partnerships further amplify resources, such as the U.K.’s National Health Service (NHS), collaboration with Cancer Research U.K. to fund community outreach, while tax-based financing models (e.g., tobacco levies) sustain long-term programs (43). These systemic investments have reduced late-stage diagnoses by 30% in some HICs, demonstrating the efficacy of integrating policy, funding, and education (44).

### 3.3 Educational resources and methods

Cancer education and prevention in LMICs face significant resource constraints, relying heavily on community-based approaches and international support. Many African nations, for example, depend on lay health worker training programs, such as Uganda’s cervical cancer network, which increased screening rates by 40% through peer-led education (45). Educational materials are often adapted for low-literacy populations, including visual pamphlets in Ethiopia that doubled screening participation (46). However, formal training programs remain scarce, with only 13 of 26 surveyed African countries receiving International Atomic Energy Agency (IAEA) support for radiotherapy education, and most medical physics training being supplier-provided, short-term (1–2 weeks), and focused on equipment operation rather than comprehensive clinical skills (47). Digital interventions are emerging but face infrastructure challenges; Kenya’s SMS-based mHealth program achieved 82% coverage, yet such initiatives remain exceptions rather than norms (48). Cultural adaptation is critical, as seen in Mexico’s success decoupling HPV vaccine education from sensitive topics, but funding instability and urban–rural disparities persist, with rural areas in countries like India having <15% mammography screening rates (49). The Middle East, particularly the United Arab Emirates (UAE), demonstrates emerging but uneven progress in cancer education amidst unique regional challenges, such as rural areas lacking the infrastructure sustaining comparable HICs’ telehealth systems (50).

Developed countries possess abundant educational resources and advanced methods for cancer education. For example, the United States provides extensive cancer education resources and online courses through institutions like the NCI and the Cancer Information Service (CIS) (51). Japan promotes cancer prevention knowledge through community and school health education (52). In comparison, developing countries have relatively limited educational resources and rely on traditional methods.

### 3.4 Public awareness and participation

In LMICs, public awareness and participation in cancer education and prevention remain limited due to socioeconomic barriers, cultural stigma, and inadequate health infrastructure, with studies indicating screening participation rates below 20% in regions like sub-Saharan Africa compared to over 70% in HICs (53–55). Cultural misconceptions, such as cancer fatalism in India and Nigeria, often delay early detection, compounded by gender norms that restrict women’s access to screening in conservative communities (56). Grassroots initiatives, such as Uganda’s peer-led cervical cancer education (40% screening increase) and Ethiopia’s faith-based outreach (tripled participation), demonstrate the potential of localized, culturally adapted campaigns (57). Urban–rural health disparities persist, particularly in LMICs, such as Bangladesh, where rural populations often depend on community health workers—exemplified by the BRAC model—due to a scarcity of clinical facilities. While mobile health (mHealth) interventions, such as the SMS reminder system in Kenya, demonstrate significant potential with an 82% reach, they encounter scalability challenges that are contingent on sustained funding (58–60).

HICs leverage robust public health campaigns, policy mandates, and digital platforms to achieve cancer awareness rates exceeding 80%, exemplified by the U.S. Pink Ribbon movement (70% mammography screening adherence) and Australia’s HPV vaccination program (85% school-based uptake) (61, 62). Government-funded initiatives, such as Japan’s annual Cancer Prevention Week and the U.K.’s NHS mass media campaigns, normalize preventive behaviors through celebrity endorsements and employer-sponsored screenings (63). Advanced health literacy enables proactive participation, with AI-driven tools (e.g., U.K. chatbot symptom checkers) and electronic health records facilitating personalized reminders, reducing late-stage diagnoses by 30% over two decades (64). Legislative measures, including mandatory workplace screenings in Germany and tax incentives for preventive care in France, institutionalize participation, while social media amplifies reach—Sweden’s 2021 melanoma awareness campaign achieved 90% population engagement via influencer partnerships (65, 66). Despite high baseline metrics, disparities persist among marginalized groups, prompting targeted interventions like the U.S. Affordable Care Act’s free screening provisions for low-income populations (67).

### 3.5 Medical system support

In LMICs, support for cancer education and prevention faces serious challenges, such as critical shortages of oncology specialists (0.3 per million in sub-Saharan Africa compared to 30 per million in HICs), fragmented primary care integration, and inadequate infrastructure for screening and treatment (68). Access to radiotherapy remains particularly limited, with Africa having only 34% of the necessary megavoltage units, and maintenance issues often cause machine downtime (69). National health systems frequently prioritize infectious diseases over cancer control, leading to inconsistent funding; only 4 out of 26 surveyed African countries reported reliable annual budgets for radiotherapy services (70). Where cancer programs exist, they usually depend on international partnerships (e.g., IAEA training support in 13 African nations) and vertical disease-specific initiatives rather than comprehensive system-strengthening (71). Notable exceptions include Ethiopia’s task-shifting model, which trains general practitioners in oncology, and Rwanda’s integrated cancer centers with community health worker networks, though these are still limited by equipment shortages and tend to be concentrated in urban areas (72). Digital innovations such as Zambia’s tele-oncology program show promise but require stable electricity and internet, which many rural areas—home to 60% of cancer patients—lack (73).

## 4 Discussion

The systematic review highlights significant disparities in cancer education between HICs and LMICs driven by a confluence of historical, socioeconomic, and policy-related factors. Our analysis indicates that HICs benefit from well-established cancer education frameworks, underpinned by comprehensive policy infrastructures, substantial financial investments, and advanced technological resources. Conversely, LMICs grapple with systemic challenges such as fragmented policies, resource limitations, and cultural obstacles, which hinder effective cancer prevention and control.

**Figure 2:**
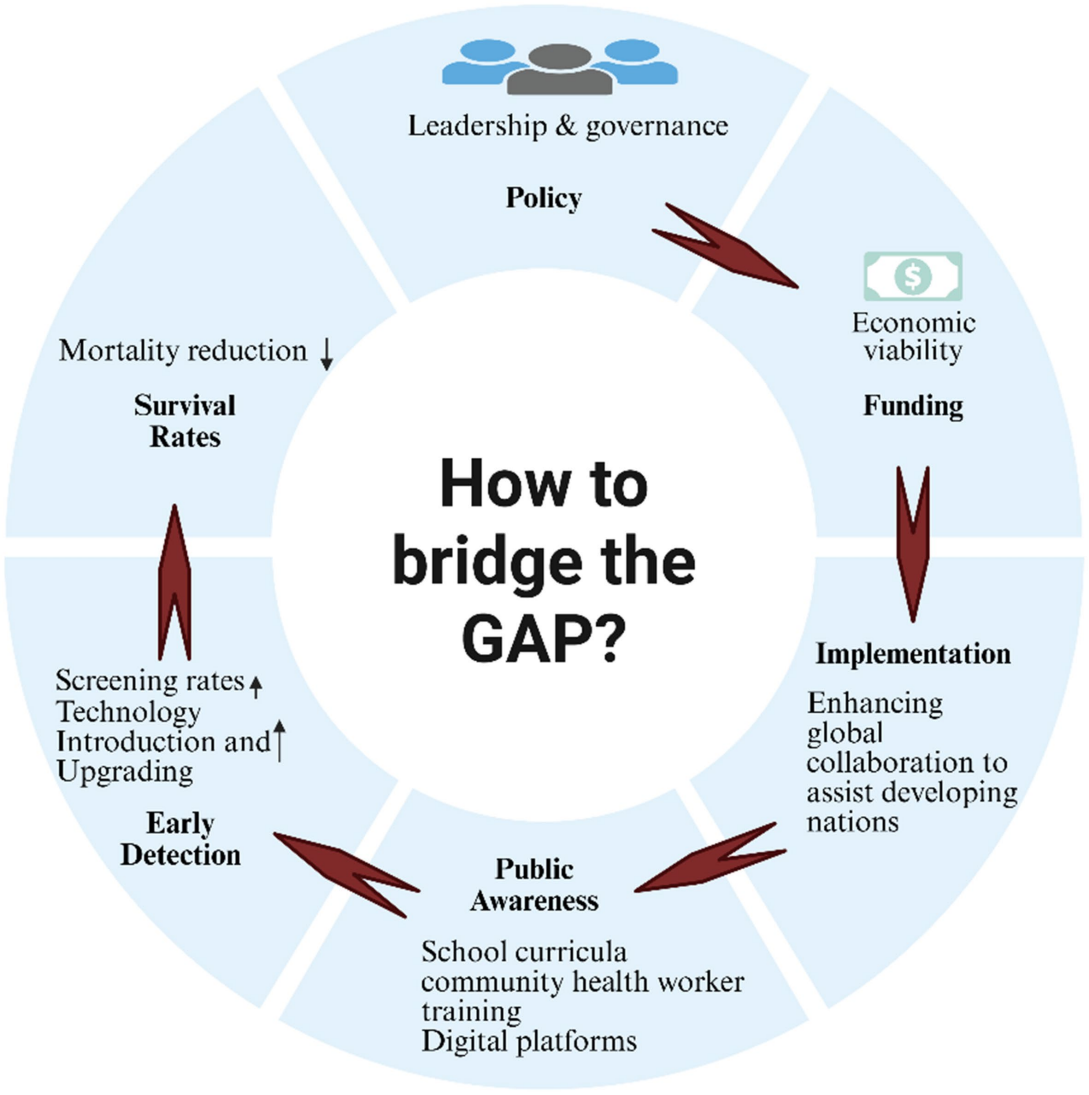
From policy to population impact: a structural visualization of cancer education implementation pathways.

This situation necessitates the development of context-specific strategies aimed at achieving health equity globally. We propose a structured framework focused on n leadership and governance in cancer control that outlines essential policy areas, including mortality reduction through enhanced survival rates and effective early detection strategies. Furthermore, critical gaps in public awareness, economic viability, and global implementation are identified, emphasizing the imperative for targeted interventions (Figure 2) Recommended strategies include the integration of school-based educational programs, the utilization of digital platforms, and fostering international collaborations to address disparities in cancer prevention and care between developed and developing nations. A comparative analysis (as shown in Table 2) underscores these disparities, revealing that while HICs exhibit comprehensive policies, higher per capita investment, and robust healthcare infrastructures, LMICs increasingly rely on community-based approaches amidst significant resource constraints. This analysis reinforces the urgent call for tailored interventions to enhance global efforts in cancer prevention and control.

**Table 2:** Comparative analysis of cancer education between developed and developing countries.

Category	Developed countries (HICs)	Developing countries (LMICs)	Key disparities
Policy Frameworks	- Comprehensive NCCPs (e.g., U.S. National Cancer Act, Japan’s Basic Law on Cancer Control) (39, 41)	- Fragmented policies; only 4/26 African countries have NCCPs (33, 37)	HICs: Legislative mandates; LMICs: Reliance on vertical/disease-specific programs
Funding (*Per Capita*)	$150–300 annually (e.g., Cancer Moonshot: $1.8B/year) (34, 35, 39)	<$10 annually; dependent on external aid (36)	15–30 times higher funding in HICs
Public Awareness	- Screening participation >70% (USA mammography) (40, 44)	- Screening <20% in sub-Saharan Africa (53–55)	HICs: Mass media campaigns; LMICs: Grassroots peer-led initiatives
Educational Methods	- Digital platforms (e.g., NCI online courses) (51)	- Community health workers (Uganda: +40% screening) (28, 45)	HICs: Technology-driven; LMICs: Low-literacy adaptations
	- School curricula (UK, Australia) (20, 24)	- Visual pamphlets (Ethiopia) (30, 46)	
Healthcare Integration	- Universal screening (e.g., NHS UK) (43, 63)	- 0.3 oncologists/million (sub-Saharan Africa) (68, 69)	HICs: Multidisciplinary care; LMICs: Task-shifting (e.g., Ethiopia’s GP training)
	- 30 oncologists/million (68, 69)	- 34% radiotherapy coverage (37, 47)	

### 4.1 Historical and policy context

The evolution of cancer education in HICs, such as the UK and the US, has been shaped by decades of policy development and institutional support. For instance, the US National Cancer Act of 1971 and subsequent initiatives like the Cancer Moonshot program have allocated billions of dollars to research and public awareness campaigns, resulting in screening participation rates exceeding 70% for diseases like breast cancer (39, 41). In contrast, LMICs, burdened by competing health priorities and limited infrastructure, have only recently begun integrating cancer education into national health strategies. Examples like Rwanda’s mandated workplace education programs and Zambia’s tele-oncology initiatives demonstrate the potential for policy innovation in resource-limited settings. However, these efforts remain constrained by funding instability and reliance on international aid, with LMICs spending less than $10 per capita on cancer education compared to $150–300 in HICs (34–36, 39).

### 4.2 Educational resources and methods

The contrast in educational resources between HICs and LMICs is stark. HICs leverage digital platforms, structured school curricula, and mass media to disseminate cancer knowledge, achieving public awareness rates above 80% (51). In LMICs, community-based approaches, such as Uganda’s peer-led cervical cancer education (which increased screening rates by 40%) and Ethiopia’s visual pamphlets (which doubled screening participation), have shown promise (28, 45). However, these interventions often face scalability challenges due to inadequate infrastructure, such as unreliable electricity and internet access in rural areas. Digital innovations like Kenya’s SMS-based mHealth program (reaching 82% coverage) highlight the potential of technology, but their sustainability depends on local capacity-building and funding (30, 46).

### 4.3 Public awareness and participation

Cultural and socioeconomic barriers significantly hinder cancer education in LMICs. In regions like sub-Saharan Africa, screening participation rates remain below 20%, compared to over 70% in HICs, due to factors such as cancer fatalism, gender norms, and limited access to healthcare. Grassroots initiatives, including faith-based outreach in Ethiopia and female-led educator programs in Pakistan, have demonstrated the importance of culturally tailored approaches (53–55). Conversely, HICs benefit from nationwide campaigns like Australia’s HPV vaccination program (85% school-based uptake) and Japan’s annual Cancer Prevention Week, which normalize preventive behaviors through policy mandates and celebrity endorsements (40, 44).

### 4.4 Healthcare system support

The integration of cancer education into healthcare systems varies widely. HICs, with their multidisciplinary care pathways and universal screening programs, achieve 5-year survival rates 2–3 times higher than LMICs. In contrast, LMICs struggle with severe shortages of oncology specialists (0.3 per million in sub-Saharan Africa vs. 30 per million in HICs) and inadequate infrastructure (43, 63, 68, 69). Task-shifting models, such as training general practitioners in oncology in Ethiopia, and hub-and-spoke networks, like Rwanda’s integrated cancer centers, offer scalable solutions but require sustained investment and political commitment (37, 47).

### 4.5 Actionable recommendations

To reduce global disparities of cancer education in the developing nations, this study proposes an integrated framework encompassing multisectoral policy development, nationwide prevention campaigns, standardized early detection protocols, and strengthened treatment networks. The approach emphasizes developing culturally adapted, evidence-based interventions while establishing robust surveillance systems and palliative care services. Crucially, it advocates for structured international collaboration to facilitate knowledge transfer and capacity building, with implementation guided by continuous monitoring to ensure adaptability to local contexts and evolving epidemiological needs. This comprehensive strategy addresses critical gaps across the cancer care continuum while optimizing resource utilization in diverse healthcare settings (Supplementary Table 3).

Based on the aforementioned considerations and summarization, this study posits that developing countries should adhere to the following strategies:

1. Policy Level: LMICs should legislate the inclusion of cancer education in primary healthcare performance indicators, drawing inspiration from Ethiopia’s Health Extension Program.
2. Content Design: Educational priorities must align with local disease burdens (e.g., esophageal cancer in East Africa) and adopt staged disclosure principles to address cultural sensitivities, as seen in Mexico’s HPV vaccine promotion.
3. Implementation: Leveraging community health workers (e.g., Bangladesh’s BRAC model) and mobile health technologies can enhance reach while minimizing costs.
4. Global Collaboration: HICs should support technology transfer (e.g., patent exemptions for essential drugs) and capacity-building initiatives (e.g., IAEA radiotherapy projects) to foster equitable progress.

## 5 Conclusion

Bridging the gap in cancer education requires a multifaceted approach that combines policy reform, localized efforts, and global cooperation. By addressing structural inequalities and utilizing innovative, culturally appropriate solutions, the worldwide community can empower individuals with knowledge, decrease stigma, and ultimately reduce the rising cancer burden in underserved areas. This goal aligns with the broader aims of universal health coverage and health equity worldwide, calling for ongoing advocacy, research, and partnership.

### 5.1 Limitations

This review has several limitations. The heterogeneity of metrics across studies complicates direct comparisons, and publication bias may overrepresent successful interventions in LMICs. While our analysis incorporates diverse regional perspectives, we recognize the persistent evidence gap regarding cancer education initiatives in North Africa and Francophone West Africa, which warrants targeted investigation in future studies. Additionally, due to limited peer-reviewed literature available in our selected databases, the current study was unable to include as many cases from Latin America and the Middle East, in the literature limits the generalizability of findings. This limitation underscores the need for expanded research efforts to better understand context-specific challenges and develop culturally appropriate interventions in these underrepresented regions and should prioritize standardized evaluation frameworks and include more diverse LMIC contexts to address these gaps.
