# Supplementary material for: Current status of cancer education in developing and developed countries: identifying the disparities and bridging the gap
Source: Front Public Health. 2025 Aug 13;13:1608525. doi: 10.3389/fpubh.2025.1608525 (PMC12380729; doi:10.3389/fpubh.2025.1608525)
Supplement: Supplementary file 1 [file Table_1.docx]

**Supplementary**

Table 3 Framework for action on cancer prevention and control in the developing nations.

| **Area** | **Strategy** | **Methods** |
| --- | --- | --- |
| Governance | Try to develop a multisectoral strategy and action plan for cancer education, as part of the national plan for cancer prevention and control. | Develop an operational, funded national multisectoral strategy. |
| Prevention | Advocate healthy lifestyle interventions in the LMICs, such as tobacco control, physical activity, healthy diet in line with the regional framework for action on cancer education. | Organize a national public awareness campaign on cancer education |
| Detection | Try to develop, implement, and update evidence-based, nationally approved guidelines for the early detection of priority cancers, with a focus on early diagnosis. | Nationwide advocacy and promotion of early cancer screening. |
| Management | Enhancing the nationwide network of cancer treatment institutions and promoting  early intervention and early treatment. | Unify and formulate cancer treatment strategies nationwide and invest in medical institutions. |
| Palliative care | Help to develop affordable, multidisciplinary, integrated palliative care services. | Enhance the availability of national  palliative care. |
| Surveillance and Research | Advocate and establish hospital- and population-based cancer registration. | Adhere to and supervise  the implementation of the  national cancer control program. |
| Cooperation | Study the cancer education policies of developed nations. | Developed nations should help the developing nations. |
