# Supplementary material for: Current status of cancer education in developing and developed countries: identifying the disparities and bridging the gap
Source: Front Public Health. 2025 Aug 13;13:1608525. doi: 10.3389/fpubh.2025.1608525 (PMC12380729; doi:10.3389/fpubh.2025.1608525)
Supplement: Supplementary file 2 [file Table_2.docx]

**Appendix**

**1. Web of Science Core Collection (WOSCC)**

**Search String:**

TS= ("cancer education" OR "oncology education" OR "cancer literacy" OR "cancer awareness") AND TS= ("developing country" OR "low- and middle-income countries" OR "LMIC" OR "low-income country" OR "developed country" OR "high-income country" OR "HIC").

**Filters:** Publication years: 2004–2024; Document types: Article/Review.

**2. PubMed/MEDLINE**

**Search String:**

(("cancer education"[Title/Abstract] OR "oncology education"[Title/Abstract] OR "cancer literacy"[Title/Abstract] OR "cancer awareness"[Title/Abstract]) AND ("developing country"[Title/Abstract] OR "low-income country"[Title/Abstract] OR "LMIC"[Title/Abstract] OR "developed country"[Title/Abstract] OR "high-income country"[Title/Abstract] OR "HIC"[Title/Abstract]))

**Filters:** Publication date: 2004–2024; Article types: Systematic reviews, Meta-analyses, Original research.

**3. Cochrane Library**

**Search String:**

TS= ("cancer education" OR "oncology education" OR "cancer literacy" OR "cancer awareness") AND TS= ("developing country" OR "low- and middle-income countries" OR "LMIC" OR "low-income country" OR "developed country" OR "high-income country" OR "HIC").

**Filters:** Publication years: 2004–2024; Study types: Article, Reviews.
